# Supplementary material for: Glycerol Electro-Oxidation in Alkaline Medium with Pt-Fe/C Electrocatalysts Synthesized by the Polyol Method: Increased Selectivity and Activity Provided by Less Expensive Catalysts
Source: Nanomaterials (Basel). 2023 Mar 25;13(7):1173. doi: 10.3390/nano13071173 (PMC10096876; doi:10.3390/nano13071173)
Supplement: Supplementary file 1 [file nanomaterials-13-01173-s001.zip › nanomaterials-2272596-supplementary.pdf]

Supplementary Information

Methodology to calculate the mass of platinum in the electrodes

The platinum mass (**m_{Pt}**), in mg, was calculated with the proportions obtained in the EDS and TGA analyses. The **m_{Pt}** values were obtained using the equation:

$$\boldsymbol{m_{Pt}} = \frac{m_{cat}}{(f_{M:C})\ x\ (f_{M1:M2})}$$

$\boldsymbol{m_{cat}}$ = total mass used to prepare ink, in eppendorf;

$\boldsymbol{f_{M1:M2}}$ = mass ratio between Pt and Fe, provided by EDS analysis;

$\mathbf{f_{M:C}}$ = proportion of mass between metal and carbon, data taken from TGA analyses;

Error! Reference source not found. presents an example of m_{cat} and m_{Pt} values.

Table S1. Examples of data for ink preparation of vitreous carbon electrodes, in an eppendor containing 100 mL of isopropyl alcohol (95%) / Nafion (5%) solution.

Composition of the Synthesis	m_{cat} (mg)	$f_{Pt:Fe}$	$f_{M:C}$	Volume deposited (L)	Mass of Pt (g)
**Pt/C**	2.35	1	0.20		23.5
**$Pt_{95}Fe_{05}/C$**	2.40	0.95	0.13	5.0	14.8
**$Pt_{75}Fe_{25}/C$-hydraz.**	2.15	0.75	0.06		4.8

(A) (B)

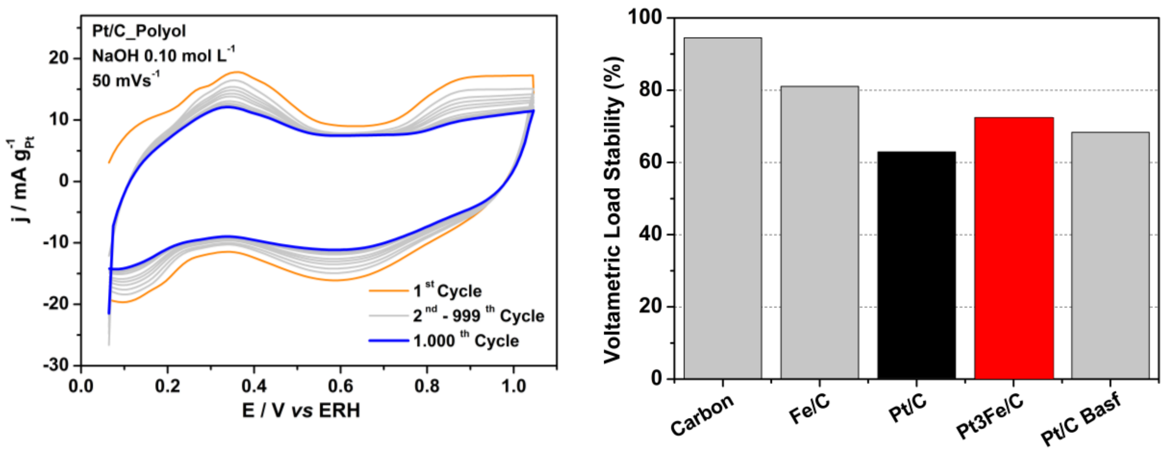

Figure S1. Stability test (**A**) and % voltammetric stability (**B**). Cycles 1000, 50 mVs^{-1}, supporting electrolyte NaOH 0.1 mol L^{-1}.
